# The Menace of *Candida auris* Epidemic Amidst the COVID-19 Pandemic: A Systematic Review

**DOI:** 10.3390/diseases10030058

**Published:** 2022-08-29

**Authors:** Hala Najeeb, Sarush Ahmed Siddiqui, Zahra Anas, Syed Hasan Ali, Shajie Ur Rehman Usmani, Fareeha Jawed, Hafsa Nazir Jatoi

**Affiliations:** Department of Internal Medicine, Dow Medical College, Dow University of Health Sciences, Karachi 74200, Pakistan

**Keywords:** COVID-19, *Candida auris*, fungal infection, pandemic, co-infection

## Abstract

Severe Acute Respiratory Syndrome Coronavirus-2 (SARS-CoV-2), the causative agent for the Coronavirus Disease 2019 (COVID-19) pandemic, has sparked a medical emergency worldwide. With the rise in COVID-19 infections and an eventual increase in hospitalized critically ill patients, a trend of bacterial, fungal, and viral superinfection has been noted. One important agent of co-infection identified is *Candida auris*. Due to its multidrug-resistant nature and easy transmissibility, *C. auris* is difficult to manage in COVID-positive patients. Patients with comorbidities, immunosuppressive states, intubated and on ventilators are more likely to contract the fungal infection. Therefore, it is essential to the first screen, diagnose, and isolate patients with *C. auris* infection and manage and treat them while preventing the spread of the disease. Failure to recognize and prevent its spread may lead to an eventual epidemic or even a pandemic during the current COVID-pandemic, which the exhausted healthcare system can most definitely not handle. This systematic review investigates the prevalence of *C. auris*, its pathophysiology, diagnosis, prevention, and treatment during the COVID-19 pandemic.

## 1. Introduction

Since its first report in China’s Wuhan Province in 2019 [1], severe acute respiratory syndrome coronavirus-2 (SARS-CoV-2) infection, the cause of the Coronavirus Disease 2019 (COVID-19), has enveloped the world in a massive pandemic. It has proudly challenged even the most advanced healthcare systems globally, leaving a lot of them in shackles. However, it has not acted alone. It has exacerbated several other healthcare burdens that were gaining increasing attention in the pre-COVID-19 world, one of which is the secondary transmission of multidrug-resistant organisms such as the fungus *Candida auris* (*C. auris*) in hospital settings in COVID-19 ICUs [2]. Secondary infections, such as those by *C. auris*, combined with a lack of natural immunity, lead to severe lung injury, acute respiratory distress syndrome (ARDS), and high mortality rates in COVID-19 patients [3].

*C. auris* has continued to make its mark across the world. This fungus has been reported on all continents of the world except Antarctica [4] since it was first discovered in a patient’s ear canal in Japan in 2009. In India, a study spanning four months showed a 60% case-fatality rate of COVID-19 patients, among which a shocking two-thirds had been diagnosed with *C. auris* infection [5]. An alarming finding from Mexico revealed a high mortality rate of over 83% in patients with COVID-19-associated *C. auris* bloodstream infection, despite antifungal therapy [6]. In four healthcare institutions in the *C. auris* endemic region of Northern Colombia [7], six (3.33%) out of 20 cases of fungemia were reported in hospitalized patients with SARS-CoV-2 from June to September 2020, who had *C. auris* infection. Various major outbreaks of *C. auris* bloodstream infections have also been reported in India, the U.K., Colombia, South Africa, and the USA These cases are nothing but the tip of the iceberg pointing toward a much larger issue than anticipated. It is, thus, imperative to identify patients quickly and correctly with *C. auris* if we hope to contain its spread [5].

Fungal infections can complicate the diagnosis, treatment, and progression of COVID-19. Established data from past coronavirus outbreaks (SARS-CoV and MERS) have demonstrated that systemic fungal infections, such as invasive aspergillosis and candidemia, have contributed to severe outcomes for I.C.U. patients [8]. Hence, it is no surprise that *C. auris*-associated superinfections have been associated with alarmingly high 30-day mortality rates in critically ill COVID-19 patients, usually above 50% [9]. It has exponentially exacerbated the burden on already saturated healthcare systems due to the ongoing COVID-19 pandemic.

*C. auris* patients continuously shed viable yeast cells from their skin and contaminate hospital environments, especially I.C.U.s that house critically ill patients. Unfortunately, I.C.U. teams in developing countries have particularly faced the challenge of over-occupancy of beds and relatively increased compromised infection prevention practices, encouraging *C. auris* to thrive and spread quickly. It is also noteworthy that several risk factors are shared between COVID-19 and *C. auris* patients in I.C.U.s, such as diabetes mellitus and chronic renal disease, enabling *C. auris* to harbor itself alongside SARS-CoV-2, forming a potentially lethal force to be reckoned with.

Despite its widespread emergence, there is still an alarming lack of identification strategies available globally, which mandates an immediate need for effective therapeutic strategies against this pathogen [10]. The lack of identification strategy also accounts for high misidentification rates due to difficulties in correctly identifying *C. auris* strains. However, that is not all. This, unbearably, is just one of the four troublesome aspects associated with *C. auris*, the other three being its high rate of antifungal drug resistance [11], its ability to colonize the skin and other bodily sites, and its aptitude to live on abiotic surfaces and equipment for weeks [12], thus explaining the mode and speed of its transmission among healthcare workers and immunosuppressed hospitalized patients [13].

The Centre for Disease Control and Prevention (C.D.C.) in the USA has recommended strict isolation of patients colonized with or treated for *C. auris*. However, the pandemic has made it severely challenging to implement these guidelines as hospital settings are already excessively overwhelmed and overburdened.

Therefore, in this systematic review, we aim to discuss the prevalence of *C. auris*, and how its pathophysiology, diagnosis, prevention, and treatment differ or are to be modified with a SARS-CoV-2 coinfection.

## 2. Methods

This systematic review was carried out along the Preferred Reporting Items for Systematic Reviews and Meta-Analyses (P.R.I.S.M.A.) guidelines statement [14]. An extensive literature search was conducted using electronic databases such as PUBMED/MEDLINE, Google Scholar, and Scopus, from inception till February 2022. The following keywords were used as a search string: (“COVID-19” or “SARS-CoV-2”) AND (“*Candida auris*” or “Candidemia”). Additional search strings included: (“COVID-19” or “SARS-CoV-2”) AND (“*Candida auris*” or “Candidemia”) AND (“Pathophysiology” OR “Clinical Manifestations” OR “Geographical Distribution” OR “Diagnosis” OR “Management” OR “Treatment”). Data extracted include title, study type, study duration, region, the prevalence of cases, clinical presentation, diagnostic techniques, and mortality, as presented in Table 1.

## 3. Results

**Figure 1:**
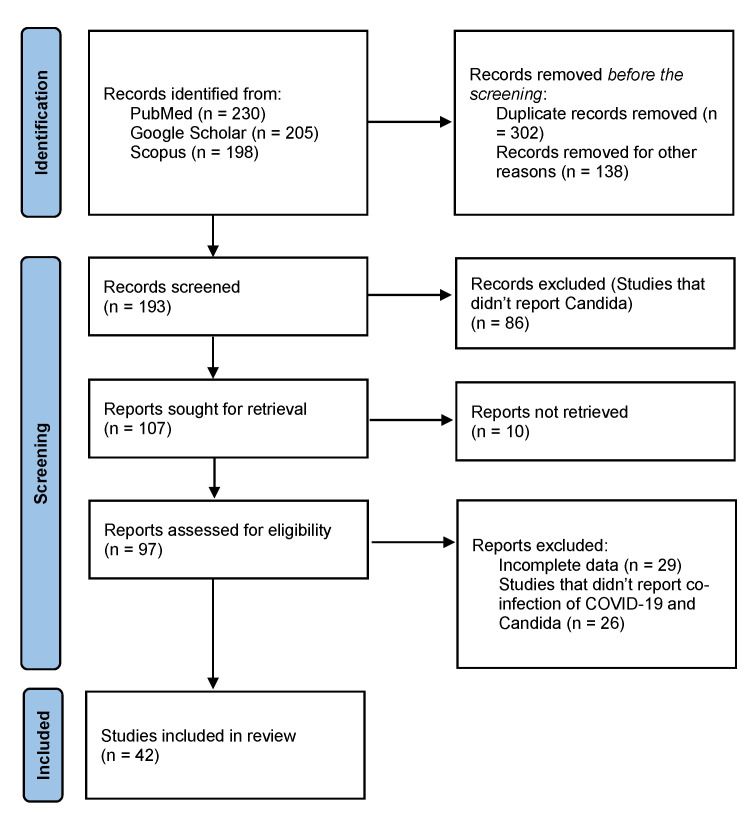
PRISMA Flowchart.

The search yielded 633 results, of which 42 studies were included, as shown in Figure 1 The search results applied no filters or limitations to date, time, location, or study type. Studies that did not report candida infection and co-infection of COVID-19 and candida were excluded. Case reports, case series, observational studies, retrospective studies, and systematic reviews which reported co-infection of COVID-19 with Candida were included in this study. Hand-searching of review articles was performed to extract relevant studies. Relevant studies were imported to Endnote X9 (Clarivate Analytics, Philadelphia, PA, USA) to remove duplicates further. The included studies comprise 5 letters-to-the-editors (LTEs), 12 original articles, 9 observational studies, 11 case reports, 1 case-control study, 3 retrospective cohort studies, and 1 systematic review.

## 4. COVID and Co-Infections

Previous studies have established that viral illnesses can lead to microbial co-infection, as observed in MERS, SARS, and influenza epidemics. The emergence of recent data in the present SARS-CoV-2 pandemic indicates an increased incidence of microbial co-infection in hospitalized COVID patients [15]. The coinfection rate ranges from 7% in hospitalized patients to 14% in critically ill patients [16]. The rate of hospital-acquired infection in COVID-19-positive patients admitted to the I.C.U. is higher because they receive invasive procedures such as intubation, mechanical ventilation, and extracorporeal membrane oxygenation that can allow microbiota to grow in instruments used in invasive procedures, allowing the hospital flora to invade the systems of critically ill patients [17].

The co-infection can primarily result from the immune dysregulation found in individuals diagnosed with COVID-19. Recent studies regarding COVID 19-associated immune dysfunction identify lymphopenia and immunosuppression due to leukocyte apoptosis and T-cell exhaustion, paving the pathway for secondary or concurrent bacterial, fungal, and viral infections [15,18]. Co-infections complicate disease progression, resulting in poor prognosis, diagnosis difficulty, and treatment with increased severity, and mortality [19]. We will further discuss in detail different fungal organisms other than *Candida auris* along with some light on bacterial and viral organisms causing infection in COVID-positive individuals to navigate how they exacerbate COVID -19 infection, the role of hospital flora in co-infection, and antibiotic/antifungal resistance.

Bacterial co-infection among respiratory viruses is common because viral pneumonia weakens the immune system and makes individuals susceptible to concurrent superinfection. Several studies have observed an increased rate of bacterial coinfection in SARS-CoV-2-positive patients suspected of sepsis, community or hospital/ventilator-associated pneumonia, bacteremia, urinary tract infection, and infective endocarditis [17,20,21,22,23]. A single-center study conducted in New York observed around 152 COVID-19 patients that received central venous catheterization also showed signs of bacteremia [24]. Another study with a similar aim stated that about 392 (1.2%) COVID-19 positive individuals had laboratory-confirmed microbial co-infection showing single and multiple pathogens in the culture. Out of which 171 COVID-19 patients suspected bacterial infection when more than one pathogen had been identified from different body sites. The most predominant bacteria found were gram-negative; *Haemophilus influenzae,* followed by *Staphylococcus aureus* [25].

Another factor contributing to bacterial growth is the widespread use of antibiotics resulting in antibiotic resistance in COVID-19 patients, considering that patients who received antibiotics 30 days before their positive microbiological cultures suffered bacterial co-infection from multi-drug resistant strains [9].

The *Candida auris* outbreak during the SARS-CoV-2 pandemic has raised a concern about other fungal species also causing co-infection in this population. The previous data concerning fungal infections shows that the immunocompromised population is more susceptible to fungemia. The SARS-CoV-2 positive patients in critical condition, requiring invasive mechanical ventilation and a prolonged hospital stay, are optimum hosts to opportunistic fungal infection. A study showed 10% fungemia in SARS-CoV-2 positive individuals with a mortality rate of 70.4%, which was higher than the mortality rate observed in bacterial co-infection [26]. In Iran, about 5% of COVID-19 patients with immunocompromised status showed oropharyngeal thrush suggesting opportunistic yeast infection in the oral mucosa. However, a similar yeast infection is also observed in individuals with H.I.V. infection, marking the role of immunosuppression in fungal co-infection [27]. During the second wave of the SARS-CoV-2 pandemic, the antifungal consumption rate increased to 15% in the entire hospital and 75% in the Intensive Care Unit. The highly consumed antifungals were echinocandins (caspofungin), azoles (fluconazole, voriconazole), and Amphotericin B (a second-line drug) in the Intensive Care Unit where most patients suffered from *C. auris* associated candidemia. Even though there was an increment in fungal co-infection and antifungal consumption, a few antifungal resistant strains were isolated. Such as *Fluconazole-resistant* *C. auris*, *echinocandin-resistant* *C. auris*, and *caspofungin-resistant* *C. dubliniensis* [27,28].

As SARS-CoV-2 damages the respiratory epithelium by decreasing mucociliary clearance that favors other opportunistic respiratory viral infections [19]. Mussuza et al. also reported a viral co-infection rate of 10% and viral superinfection of 4% when additional RT-PCR assay tests showed different viruses other than SARS-CoV-2 [29]. Seasonality may play its role in viral illness outbreaks because respiratory viruses other than COVID 19 such as influenza are common in the cold season and may result in variations in viral co-infection rates. About 22.3% of COVID-19 patients had influenza A virus, and 19.3% were alive individuals. The higher mortality was associated with the seasonal outbreak, bacterial superinfection, comorbidities, and a lack of vaccination against influenza in that area [15,26,30]. These viruses usually cause flu-like symptoms such as cough, sore throat, fever, rhinorrhea, dyspnea, lung infection, and splenomegaly that can exacerbate the COVID-19 symptoms [19,30].

Table 2 highlights all the microbial species causing co-infection with their specific symptoms in COVID-19 positive patients [16,17,19,29,30,31,32,33,34].

## 5. Types of Invasive Mycoses

SARS-CoV-2 is aggressive towards lung parenchyma, making respiratory epithelium vulnerable to opportunistic invasive fungal infections (I.F.I.) such as invasive candidiasis, invasive aspergillosis, cryptococcosis, and mucormycosis [36,37]. Usually, these fungi are associated with immunocompromised individuals with an underlying cause such as T-cell exhaustion in severe COVID-19 infection, tumors, H.I.V., organ transplantation, and other comorbidities. However, I.F.I. also affects immunocompetent individuals [38]. These fungal infections usually enter the bloodstream and cause systemic inflammation [31]. Kula et al. reported Invasive Mold Disease in patients with fatal COVID-19 indicating 2% of individuals with evident invasive pulmonary aspergillosis, mycosis, and disseminated mucormycosis in eyes, and sinuses, lungs, hilar lymph nodes, brain, and kidneys. These individuals received IL-1 inhibitors, IL-6 inhibitors, and corticosteroid therapy which predisposed them to invasive mycoses [28,39].

The yeast infection and COVID-associated pulmonary aspergillosis (CAPA) were prevalent in 26.7% of individuals positive for SARS-CoV-2. The combined mortality rate for both Invasive Fungal Diseases was 52.8%, which increased without proper antifungal treatment [40]. In a single-centered prospective study, 3.3% of 239 non-immunocompromised individuals presented with COVID-associated pulmonary aspergillosis (CAPA). Those who developed acute respiratory distress syndrome (a complication of COVID-19 infection) received tocilizumab and mechanical ventilation which may have predisposed them to invasive pulmonary aspergillosis [41]. In Chile, 11% (16) I.C.U. admitted SARS-CoV-2 positive patient COVID-associated invasive mold infection (C.A.I.M.I.). The patients were diagnosed with mold infections from non-aspergillus species such as Rhizopus and scedosporium species [42]. Certain invasive mycoses such as *coccidiomycosis, blastomycosis,* and *histoplasmosis* have similar COVID symptoms. Hence, they can mimic COVID 19 infection [38]. Table 3 summarizes the opportunistic invasive mycoses causing invasive fungal diseases in COVID-19 patients [36,40,41,42,43,44].

## 6. Epidemiology

With the increasing incidence of fungal and yeast infections globally, *C. auris* was first detected in 2009 in a patient from Japan after isolating an ear sample [45]. Since then, 30 Candida species have been identified [46]. Of all the Candida species, the multi-drug resistant *C. auris*, with its easy transmissibility, emerges as a rising global challenge [47]. According to a study [48], phylogenetic analysis from 54 isolates of *C. auris* from four regions around the world showed four major clades of the fungus separated by tens of thousands of single nucleotide polymorphisms (SNPs), each of which is localized to distinct geographical locations, hence supporting the hypothesis that these clades emerged simultaneously and independently in separate geographical human populations. It remains the second most prevalent species of Candidemia [49] and requires immediate action to control its spread.

At present, the spread of SARS-CoV-2 has curated breeding grounds for *C. auris* in immunocompromised patients. The deadly combo of COVID-19 and *C. auris* spreads in hospital settings through humans and inanimate surfaces [50]. While personal protective equipment has reduced human-to-human transmission, cross-contamination through equipment remains an unavoidable phenomenon [5]. The use of equipment without proper disinfection exacerbated the coronavirus pandemic when hospital facilities were already overburdened. Oxygen tubing, C.T. scans, X-ray machines, and reusable axillary thermometers are suspected of being used for multiple patients without disinfection [50]. Cultures collected from hospital bed railings, floor, call buttons, and bins tested positive for *C. auris* [5].

Until January 2022, COVID-19 associated with *C. auris* has affected over ten countries, including but not limited to Mexico, Italy, Lebanon, USA, China, and Brazil. This fungal infection has affected over 1010 individuals worldwide, with at least 75 deaths. The USA Spain, Colombia, and Italy rank on the top charts with 179, 166, 148, and 111 cases respectively. The case series in Lebanon [51] reported five deaths, one resulting from respiratory failure. Table 3 summarizes the regions, clinical manifestations, diagnostic tools, and risk factors of *C. auris* in coronavirus patients.

## 7. Pathophysiology

### 7.1. Pathophysiology of SARS-CoV-2

The pathogenesis of SARS-CoV-2, the COVID-19-causing pathogen, is complicated and not fully understood. However, with the current literature, effective therapeutic strategies are constantly being worked upon and introduced, which is a positive indicator of progress and a reminder that more research is needed to combat this pandemic actively.

SARS-CoV-2, upon entering the body via the respiratory pathway, acts on ACE-2 receptors and releases its genetic material (R.N.A.) inside epithelial cells of the airway. There, it multiplies and is then released back out, further infecting neighboring cells and entering the alveolar area (respiratory zone) of the lung [52]. After binding with the ACE-2 receptor inside the airway epithelial cells, the SARS-CoV-2 triggers localized inflammation, endothelial activation and damage, tissue damage, and cytokine release, thereby participating in the pathophysiology of acute respiratory distress syndrome (ARDS). It is noteworthy that many COVID-19 patients died due to ARDS [53]. Furthermore, the endothelial damage may also trigger Disseminated Intravascular Coagulation (D.I.C.). As a result of the D.I.C. and congestion of the small capillaries by inflammatory cells, there is a risk of possible thrombosis in larger vessels, causing lung ischemia, which may stimulate angiogenesis and epithelial cell hyperplasia [54]. To make matters worse, the virus invades the bloodstream from the respiratory tract (by infecting and damaging epithelial cells). It disseminates throughout the body to organs including the heart, brain, kidney, liver, and gastrointestinal tract, causing multiple extrapulmonary manifestations such as cerebral hemorrhage, ischemic stroke, paralysis, coma, and possibly, death [55]. 

The enhanced vascular permeability and leakage in patients with severe COVID-19 may happen due to multiple proposed mechanisms [56]. As the virus binds the ACE-2 receptor to enter the host cells, it reduces the receptor’s activity and indirectly triggers the kallikrein-bradykinin pathway, leading to increased vascular permeability. Also, the damaged epithelial cells recruit neutrophils, which, when activated, produce cytotoxic mediators including reactive oxygen species (R.O.S.) [56]. The ‘cytokine storm’, propagates the damage progression and leads to other epithelial cell dysfunction, inflammation, D.I.C., and vasodilation of the pulmonary capillaries. Altogether, this combination of events ultimately leads to multi-organ failure and death (due to alveolar dysfunction and ARDS with hypoxic respiratory failure) seen in patients with severe COVID-19.

### 7.2. Pathophysiology of Candida auris

*Candida auris,* usually a hospital-acquired fungus, has been found in several infection sites throughout the body, including urine, bile, blood, wounds, nares, axilla, skin, and rectum-infected individuals (reviewed by [57]). *C. auris*, unlike *Candida albicans*, does not primarily colonize the gastrointestinal or genitourinary tracts of healthy individuals. Instead, it is thought to colonize the skin primarily and predominantly. It has rarely been isolated from the oral, esophageal, and gut mucosa of infected individuals [48]. A study showed how in-vivo experiments and clinical manifestations together suggest that *C. auris* is incapable of colonizing anaerobic body environments, such as the gut [58]. Even in the oral mucosa, histatin-5, a salivary antimicrobial peptide, has been shown to have a potent antifungal effect on *C. auris*, hence limiting its colonization [59].

In clinical settings, *C. auris* is most associated with bloodstream infections [60]. *C. auris* has been observed to cause infections ranging from mild cutaneous otitis to CSF meningitis. The very first case of *C. auris* reported in Iran was a female with otomycosis. The candida strain was identified from external ear canal discharge [61]. A pediatric patient in Iran was reported to have candida-associated meningitis. Thereafter, the number of CSF meningitis caused by *C. auris* increased with reports coming in from the UK, India, and Iran [62] *Candida auris* has been shown to cause infections in patients of all ages and genders but it has been reported to predominantly affect male patients and critically ill patients in ICUs [63]. In terms of pathogenic potential, *C. auris* has shown to be less virulent than *C. albicans, but* significantly more virulent than *C. glabrata* and *C. haemulonii* in animal models [64].

The delayed occurrence of candidemia in patients is suggestive of nosocomial transmission [63].

In terms of virulence attributes, there are many similarities between *C. auris* and *C. albicans*, including their tissue invasion, enzyme secretion, nutrient acquisition, histidine kinase-2 component system, multi-drug efflux, genes, and pathways involved in nutrient acquisition and cell wall modeling [65]. A study performed on a *C. auris* isolate from a case of vulvovaginitis showed proteinase, phospholipase, and haemolysin activity [66]. Another study showed that the production of proteinases and phospholipases is strain-dependent [67]. Even though most strains of *C. auris* form biofilms, there are reports of a lack of biofilm formation in some strains [65,67,68]. In-vitro studies on isolates of *C. auris* have shown that isolates can be phenotypically divided into aggregating and non-aggregating strains [68], with the aggregative strain having the capability not to be physically disrupted, even by detergent treatment or by vortexing [69]. This property may contribute to the survival of *C. auris* isolates in hospital environments. The non-aggregating isolates, on the other hand, have shown to exhibit more pathogenicity than aggregating isolates and similar pathogenicity to *C. albicans* [69]. Some reports have also suggested that these non-aggregating isolates can be more pathogenic than *C. albicans* [68]. However, the exact pathogenesis of *C. auris* is still poorly understood and understandably requires research.

## 8. Risk Factors

The coronavirus’s most striking characteristic is its spike protein’s ability to attach to the ACE-2 receptors in the lungs, which downregulates its expression and overproduces angiotensin-2, leading to lung injury. Additionally, activating macrophages by the antigen presentation cells produces a ’cytokine storm’ of CCL2, IFN-α, IFN-γ, and IL-6 [69]. Diarrhea and vomiting follow the infection as pro-inflammatory cytokines disrupt the mucosa. This provides a route of entry for fungal infections such as candidemia which spread from the gut to the lumen [70]. COVID-19 patients reportedly have lower expression of CD4+ and CD8+ T-cells. COVID-19 patients have a lymphocyte count below normal, accounting for lymphocytopenia [71]. Overproduction of pro-inflammatory mediators, specifically IL-6, associates them with a worse prognosis of COVID-19 and puts them at risk for opportunistic infections [31].

While there is no specific treatment for COVID-19, antifungals, broad-spectrum antibiotics, and corticosteroids are being used to treat mild to severe cases [72]. Tocilizumab, an IL-6 receptor monoclonal antibody, has clinically reduced damage to the immune system. While the promising results show decreased oxygen requirements in severe COVID-19 patients [73], it increases the susceptibility of candidemia in such immunocompromised patients as their innate immune response to fungi and viruses is suppressed [74] Similarly, corticosteroid therapy such as hydrocortisone and dexamethasone suppress the immune system, posing a threat for superimposed fungal infections such as candidemia. Meropenem or moxifloxacin, broad-spectrum antibiotics, are frequently used in severe coronavirus patients. This allows oropharyngeal candidemia to cross barriers through the disrupted mucosal wall [75] A study in Iran showed that using wide-spectrum antibiotics in COVID-19 patients was the leading risk factor of oropharyngeal candidemia in 92.5% of patients [76].

An impaired immune response to COVID-19 is further endangered in the presence of underlying comorbidities, the most threatening being respiratory illnesses, diabetes, chronic kidney disease, and cancer. Hyperglycemia, lymphocytopenia, and inflammatory cytokines create a perfect storm for opportunistic candidemia. In non-COVID circumstances, chemotherapy and cancer accounted for one-third of the candidemia cases [31,77]; while non-clinical risk factors of *C. auris* include the patient’s age and sex. A retrospective study from India reported that COVID-19 patients above the age of 60 and the male sex were associated with a candidemia infection [78].

A worse prognosis of COVID-19 manifests as moderate to severe pneumonia, which requires I.C.U. the admission followed by mechanical ventilation in critical cases. The median stay of COVID-19 patients at an intensive care unit ranged from one to three weeks [79]. Transfer of patients from hospitals or areas endemic with candidemia, previous I.C.U. admission, prolonged hospital stays, and invasive procedures such as intubation and indwelling central lines and urinary catheters may bring the patient in contact with the hospital environment, which poses a risk of developing candidemia infection through cross-contamination [78,80]. As the rising coronavirus cases burdened the healthcare system, many facilities were scrambling for resources. This became evident as improperly disinfected axillary thermometers became increasingly common. Similarly, a study in Brazil reported positive cultures for reusable probes and bed railings in a COVID-19 setting [5]. Table 4 gives a summary of clinical and non-clinical risk factors of *C. auris* in COVID-19 patients.

## 9. Clinical Presentation and Complications

COVID-19 caused by the severe acute respiratory syndrome coronavirus 2 (SARS-CoV 2) is predominantly a respiratory illness, with clinical signs and symptoms that may vary from person to person. Some patients remained asymptomatic with a self-limiting illness throughout the disease, while some showed visible symptoms [86]. The most frequently seen symptoms included fever, cough, fatigue, expectoration, anorexia, sputum production, and shortness of breath. Headache, sore throat, confusion, and hemoptysis were some of the less commonly seen symptoms [86]. The clinical presentation of the patient depends on the severity of the illness. In severe cases, patients experience complications including acute respiratory distress syndrome, sudden cardiac death, liver dysfunction, and multi-organ failure [87].

Superimposed fungal infection in a COVID-19 patient can exacerbate the severity of the illness. A worrisome complication of COVID-19 was seen with the emergence of a fungal co-infection that caused COVID patients to suffer from candidemia. Laboratory reports of about 85% of the COVID patients showed lymphopenia making them more vulnerable to opportunistic nosocomial infections [87]. *C. auris* outbreaks tend to occur in critically ill hospitalized patients with mortality rates as high as 72% [4]. The mortality rate of COVID-19-associated candidemia (C.A.C.) remained high even after treatment with antifungal drugs [9,88]. There is limited data for the symptomatic presentation of a COVID-19 patient co-infected with *C. auris*. However, based on a few case reports, it was observed that the patients who eventually succumbed to fungal infection by *C. auris* presented to the hospital with complaints of progressive dyspnea, fever, and cough, all indicative of COVID-19 and had to be given critical care [89]. They were assisted with mechanical ventilation to maintain their oxygen saturation, and were given steroids, antibiotics, and an extended hospital stay [87,89,90]. All these factors made them vulnerable to colonization and infection by *C. auris*. It was observed that even after the resolution of coronavirus, the patients remained critically ill and could not maintain their oxygen saturation. One of the patients diagnosed with fungal co-infection by *C. auris* on day 20 of admission died due to cardiorespiratory arrest [89]. Since fungal growth was detected in their blood cultures, it can be thought that candidiasis was the cause of debilitation in these patients [89,90].

A variety of invasive fungal infections have been associated with *C. auris* [57]. Invasive candidiasis is a worrisome infection that can affect the brain, heart, blood, ears, eyes, bones, and other body parts [91]. The most feared and common complication of invasive candidiasis is bloodstream infection; candidemia [91]. A potentially fatal condition presents with a fever that does not subside with antibiotics, chills, pains, redness, swelling, and a general feeling of malaise and fatigue [92]. Candidiasis is also associated with respiratory and urinary tract infections, ear canal, and pericarditis [57]. A case report by Breazzano et al. suggested a link between endogenous panopthalmitis and *C. auris*. The histopathological analysis of the ocular tissue layers of this patient showed massive polymorphonuclear infiltration. The patient’s ocular specimens showed that he was infected with *C. auris* and Pseudomonas aeruginosa. Due to the lack of literature on the association of panopthalmitis with fungal and bacterial infections the cause of panopthalmitis in this patient remains uncertain [93]. However, *C. auris* could be highly suspected of causing panopthalmitis in immunocompromised patients [94]. Spondylodiscitis can also be a rare complication of *C. auris*. The first report published on the association of spondylodiscitis with *C. auris* suggested that about 1% of all reported spondylodiscitis cases are associated with Candida species [95]. With the increasing spread of *C. auris* and simultaneous rise in otologic infections, it was reported that this fungal isolate can also be responsible for causing fungal otomastoiditis, a rare disease of immunocompromised patients [96,97]. *C. auris* is an opportunistic pathogen that causes opportunistic infections. Some of the infections observed in a patient in Belgium include vulvovaginitis, pleuritis, intra-abdominal infections, pericarditis, ventriculitis, surgical wound infections, and osteomyelitis [97].

Admitted COVID patients presenting any of the signs mentioned earlier/infections or consistently deteriorating in health regardless of the provided treatment should be suspected of fungal co-infection by *Candida auris* and immediately screened for early diagnosis and effective treatment.

## 10. Diagnosis

Proper diagnosis of any disease is necessary for adequate treatment and prevention interventions. The diagnosis of candidiasis can be achieved by using the culture method, which includes fungal culturing of blood or other body fluids (urine, CSF, Perineal fluid) or pus from the affected site, as well as swab surveillance samples were taken to detect skin colonization from the axilla, groin, rectum, vagina, nares, and oropharynx under sterile conditions [98,99]. Although the culture method is the gold standard for diagnosis, sometimes invasive candidiasis cannot be diagnosed due to the low concentration of yeast cells in the blood or tissue organs. Therefore, non-culture diagnostic tests have also been introduced as adjuncts to cultures, including mannan and anti-mannan IgG tests, B.D.G., and PCR-based assays [100].

*C. auris* can be identified by various techniques, namely, phenotypic/culture, commercial biochemical, and molecular methods [101]. The phenotypic/ culture method identifies this isolate based on the appearance and color of colonies formed in various culture broths [102]. On culturing *C. auris* in a Sabouraud dextrose/glucose agar, smooth and white/cream-colored colonies are seen. Whereas isolates of this species, when cultured on commercial chromogenic candida agar medium, produce pink, beige, red, or pale rose-colored colonies [103]. The sabouraud dextrose agar has a sensitivity of 100% and a specificity of less than or equal to 100% [101]. Another culture broth formed by combining two culture media, CHROMagar candida media supplemented with Pal’s medium, gave a sensitivity and specificity of 100% and was best to differentiate between *C. auris* and Candida Haemulonii. *C. auris* isolates produced white-cream colonies at 420 C, whereas C. haemulonii isolates produced light pink colonies and stopped growing at 420 C [101]. However, these methods cannot solely be used to identify *C. auris* since it is difficult to distinguish it from other candida species without other techniques [102].

Traditional biochemical methods of identification, including API 20C, Vitek 2 (bioMérieux, Marcy-l’Étoile, France), Phoenix (B.D. Diagnostics, Ukraine), and MicroScan (Beckman Coulter, Pasadena, CA, USA), are known to misdiagnose *C. auris* as other yeasts [57]. This isolate confirms identification is made using accepted methods such as MALDI-TOF or molecular identification methods such as PCR (polymerase chain reaction), sequencing, or A.F.L.P. (amplified fragment length polymorphism) [98].

MALDI-TOF MS technique is an accurate and reliable method for identifying *C. auris* [60]. The matrix-associated laser desorption/ionization time-of-flight mass spectrometry (MALDI-TOF MS) method is available in two platforms, namely, Bruker biotyperTM, which is more commonly used than the VITEK MS platform [101]. The *C. auris* isolate can be identified with 100% specificity within a few minutes using this technology [101]. The MALDI-TOF method compares the obtained spectra with a reference database that contains the *C. auris* strain spectrum [57,103]. Identification will only be possible if the ‘research use only (R.U.O.) database contains *C. auris* spectrum [57,103]. Laboratories should therefore confirm beforehand whether the used reference database or research library contains the *C. auris* strain spectrum or not [103].

The molecular method used for species identification, i.e., Conventional PCR, A.F.L.P., W.G.S., Real time-PCR, has proved to be an authentic technique for detecting *C. auris*. Genomic sequencing of the D1−D2 region of the 28 S ribosomal D.N.A. (rDNA) or the internal transcribed region (ITS) by conventional PCR and sequencing of the amplicons is currently the gold standard for identifying *C. auris* providing 100% sensitivity and specificity with a short turnaround time [101]. The sequenced amplicons of the isolate are compared with the available sequences at the GEN bank to identify the isolate as *C. auris* [101]. Therefore, the isolate is usually identified by the MALDI-TOF technique, and its presence is further confirmed by the molecular amplification and sequencing method [5].

Once *C. auris* infection is suspected, it can be diagnosed using the aforementioned techniques. However, there are certain hurdles and barriers faced by the healthcare community regarding diagnostics. Since the technology needed to identify this isolate is very advanced, resource-limited countries face a significant challenge in identifying and controlling and prevention [4]. Developing or low-income countries with limited diagnostic resources lead to the under-recognition of fungal co-infections in COVID-19 patients [4]. Furthermore, even though facilitated with all the required diagnostic technology, some healthcare setups fail to correctly identify the isolate as *C. auris* is frequently misdiagnosed as Candida haemulonii/Candida famata/Candida sake or Rhodotorula glutinis due to errors in the automated identification systems [104]. Another major problem in the diagnosis of fungal co-infection is the overlapping of clinical symptoms. Similar or overlapping physical findings, clinical symptoms, routine laboratory, and radiographic results have made differentiating between fungal and bacterial co-infections a matter of great difficulty [104]. With the severity of disease increasing in a COVID-19 patient, more frequently thought is given to bacterium or even mycoplasma as the causative co-infection agent with little or no regard given to fungal co-infections [76]. This leads to a delay in diagnosing fungal co-infection and increases the risk of mortality [76].

In a study conducted to determine the various co-infections in COVID-19 patients, it was observed that co-infection could cause a false negative result of real-time reverse-transcriptase polymerase chain reaction for severe acute respiratory syndrome coronavirus 2 (SARS-CoV-2) [105]. According to the reports, the co-infectious agent that resulted in a false negative report was the most common viral pathogen, namely, the influenza A virus [105]. Due to the lack of data concerning fungal co-infections in COVID-19 patients, nothing can be said about a false-negative COVID-19 test result during candidiasis caused by *C. auris* [106]. It is, therefore, necessary to carry out a detailed and proper investigation of the disease in case there is a doubt of co-infection. The high mortality rates in COVID-19 patients due to fungal co-infections, which are either delayed or neglected, make early diagnosis and appropriate management necessary for patient survival [87]. The French High Council has recommended systematic screening for fungal co-infection in COVID-19 patients for Public Health for early diagnosis [51]. To curb the spread of this infection, rapid detection and efficient reporting of new cases are essential [51]. COVID-19 patients who are deteriorating should be suspected of fungal and bacterial co-infection and screened without delay [79].

## 11. Treatment and Prevention

The treatment of *C. auris* infection in a COVID-19 diagnosed patient is the same as in a non-COVID-19 patient. The options of treatment comprise the following categories of antifungal agents: polyenes (amphotericin B, deoxycholate, and liposomal amphotericin B), triazoles (fluconazole, voriconazole, itraconazole), echinocandins (caspofungin, anidulafungin, and micafungin), and flucytosine [37,107].

Echinocandin is labeled as the first-line therapy for *C. auris* infection [108]. If the patient is clinically unresponsive to this treatment, combination therapy of echinocandin and liposomal amphotericin B should be prescribed as synergistic interactions have better efficacy [109]. Due to the emerging yet few cases of resistance to echinocandins, patients should undergo close follow-up and microbiological culture-based reassessment to detect the therapeutic failure and eventual development of resistance.

Treating *C. auris* infection has become difficult due to its multiresistant nature. As per a report published in C.D.C., more than 99% of the *C. auris* isolates in New York were shown to be resistant to fluconazole, and around 61% were resistant to amphotericin B, and up to 4% were resistant to echinocandins [6]. It is to be noted that a high resistance rate exists with fluconazole and amphotericin B amongst *C. auris* worldwide. In a study conducted by Magnasco et al. in Italy in 2020, 6 of 14 critically ill COVID-19 patients had a *C. auris* colonization and infection. All strains of *C. auris* identified proved to be resistant to amphotericin B and azoles but susceptible to echinocandins [110]. A low number (about 4%) of isolates show resistance to all classes of antifungals; it is the only species of Candida that has isolates shown to be resistant to all classes of human antifungal drugs [111,112].

Considering multi- and pan-resistant cases of *C. auris*, the development of other antifungals has been highlighted in medicine. Fosmanogepix (APX001), an N-phosphonooxymethyl prodrug, is an antifungal agent currently in phase 2 of clinical development to treat life-threatening invasive fungal infections. It is active against a broad range of pathogenic yeast and molds, including Candida, by inhibiting the highly conserved fungal enzyme Gwt1, which is essential for the biosynthesis of glycosylphosphatidylinositol anchors [113]. Another drug currently in phase 3 of clinical trials is ibrexafungerp (SCY-078). It is the first representative of a novel class of structurally distinct glucan synthase inhibitors with broad and potent activity against Candida species, including echinocandin-resistant strains of Candida spp [114]. In addition, Rezafungin, which is currently in phase 3 of clinical trials, is a novel once-weekly echinocandin and has also proven effective against Candida spp. and Aspergillus spp. including subsets of echinocandin-resistant *C. auris* [115].

The possibility of transmission and outbreak of *C. auris* in admitted COVID-19 patients is likely due to all risk factors, such as broad-spectrum antibacterial treatment, central venous or bladder catheters, comorbidities, and mechanical ventilation [36]. Strict prevention and control methods must be put in play by hospitals and health care facilities to control nosocomial spread. As per C.D.C.’s guidelines, patients with *C. auris* should be isolated in a single patient room with appropriate contact precautions. Regular hand hygiene should be emphasized by healthcare personnel and patients. Daily disinfection of the patient care environment should occur, and contacts of newly identified patients should be screened to identify *C. auris* colonization [116]. Common disinfectants such as quaternary ammonia compounds have proven ineffective, but surface disinfectants such as chlorine and hydrogen peroxide have good efficacy against *C. auris* [112]. With the implementation of C.D.C.’s recommendations for infection control, it is possible to prevent the spread of *C. auris* in hospital care settings, as shown in a brief report by Reimer-McAtee et al. They report the success of transmission control in in-patient rehabilitation and intensive care settings [109].

## 12. Conclusions

With the increasing cases of COVID-19 exhausting healthcare facilities around the globe, any additional outbreaks, local or widespread, will undoubtedly be a challenge to manage and resolve. Given that the breeding grounds and risk factors of Candida transmission are present in hospitals, especially in COVID-19 patients, it is crucial to highlight and recognize the possibility of *C. auris* outbreaks among these patients. The multisystem involvement, rapid progression, and failure associated with the Candida species require additional medical intervention and must be given priority. In addition, necessary preventive measures should be considered in all medical setups worldwide. It is also worth noting that due to the increased antifungal use and eventual resistance, the development and evolution of medical therapy for fungal infections should be given due importance. Such advancements in health care will help decrease the spread of Candida and the eventual mortality of patients. The healthcare sector must watch for co-morbidities, and care and devise long-term policies that facilitate patient-specific care to curb another epidemic.

## Figures and Tables

**Table 1:** Regions, clinical manifestations, diagnostic tools, and risk factors of *C. auris* in coronavirus patients.

Title	Study Type	Study Period	Region	Prevalence (% or Number)	Clinical Presentation	Diagnostic Technique	Mortality
Outbreak of *Candida auris* infection in a COVID-19 hospital in Mexico	Letter	April to May 2021	Mexico	12	Mechanical ventilation	Matrix-assisted laser desorption/ionization time-of-flight	83.30%
					Peripherally inserted central lines (PICCs)	Mass spectrometry	
					Urinary catheters	Confirmed by multilocus sequence typing	
					Prolonged hospital stay (20–70 days).		
Emergence of *Candida auris* in Brazil in a COVID-19 Intensive Care Unit	Original A	1 December 2021	Brazil	2	Case 1: fever subsided	CVCt culture identified by Vitek 2 automated system	-
					Case 2: hemodialysis catheter exit showed inflammatory signs,		
					catheter-related bloodstream infections		
*Candida auris* Outbreak in a COVID-19 Specialty Care Unit-Florida, July–August 2020	Case report	July to August 2020	USA	67	Invasive infection	Clinical cultures	40%
The lurking scourge of multidrug-resistant *Candida auris* in times of COVID-19 pandemic	Letter	-	-	4			30% to 72%
First *Candida auris* Outbreak during a COVID-19 Pandemic in a Tertiary-Care Center in Lebanon	Case report	October to December 2020	Lebanon	14	Pneumonia and respiratory failure	MS-VITEK matrix-assisted laser desorption ionization time of flight mass spectrometry	35%
					five patients expired		
Multidrug-Resistant *Candida auris* Infections in Critically Ill Coronavirus Disease Patients, India, April–July 2020	case report	April to July 2020	India	15		Cultured from blood and urine samples	60%
Spread of Carbapenem-Resistant Gram-Negatives and *Candida auris* during the COVID-19 Pandemic in Critically Ill Patients: One Step Back in Antimicrobial Stewardship?	Original Article	February to May 2020	Italy	4	two out of four patients expired	using Vitek2 system	50%
Axillary Digital Thermometers uplifted a multidrug-susceptible *Candida auris* outbreak among COVID-19 patients in Brazil	Original Article		Brazil	66		Body swabs Central venous catheter tip (CVC-tip) Blood cultures Urine Culture	33.3%
Genomic Characterizations of Clade III Lineage of *Candida auris*, California, USA	Case report	September 2019–September 2020	USA	6		Swab samplesPCR	-
Molecular Epidemiological Investigation of a Nosocomial Cluster of *C. auris*: Evidence of Recent Emergence in Italy and Ease of Transmission during the COVID-19 Pandemic	Original Article	July 2019 to May 2020	Italy	10		Matrix-assisted laser desorption ionization-time of flight mass spectrometry (MALDI-TOF MS	50%
						confirmed by PCR amplification	
Increased incidence of candidemia in a tertiary care hospital with the COVID-19 pandemic	Original Article	January 2019 to February 2020 (period 1)	Brazil	Period 1 incidence of candidemia (per 1000 admissions): 1.54 and	Diarrhea Nausea abdominal pain vomiting		61.00%
		March to September 2020 (period 2).		Period 2: 7.44			
Oropharyngeal candidiasis in hospitalized COVID-19 Patients from Iran: Species identification and antifungal susceptibility pattern	Original Article	20-Jun	Iran	65	LymLeucopeniaLeucopaenia, Leucocytosis, Prolonged fever, Respiratory distress	train identification was performed by PCR sequencing of the internal transcribed spacer region	-
The impact of the COVID-19 pandemic on healthcare-associated infections in intensive care unit patients: a retrospective cohort study	Retrospective Cohort	March to April 2020	Italy	59			2019: 10 patients expired
							2020: 35 patients expired
Prevalence of candidiasis and oral candidiasis in COVID-19 patients: a cross-sectional pilot study from the patients’ registry in a large health center	Observational	21-July		106 and 14			-
High mortality co-infections of COVID-19 patients: mucormycosis and other fungal infections	Original Article	1 October	USA	6		clinical cultures	100%
COVID-19-Associated Candidiasis (CAC): An Underestimated Complication in the Absence of Immunological Predispositions?	Original Article	20 October				clinical cultures	
Impact of the COVID-19 Pandemic on Antimicrobial Consumption and Hospital-Acquired Candidemia and Multidrug-Resistant Bloodstream Infections	Original Article	March to May 2020	Spain			blood cultures were obtained > 48 h after admission.	
ICU-acquired bloodstream infections in critically ill patients with COVID-19	Observational	March to May 2020	Greece	7		blood culture	
*C. auris* and non-*C. auris* candidemia in hospitalized adult and pediatric COVID-19 patients; single center data from Pakistan	Observational	April–December 2020	Pakistan	26		blood cultures	67%
The landscape of candidemia during the COVID-19 pandemic	Observational	April–August 2020	USA	64		culture	20.30%
Incidence and Risk Factors for COVID-19 Associated Candidemia (CAC) in ICU Patients	Observational	August 2020 to August 2021	Turkey	63	bacteremia	Isolated blood culture, urine, deep tracheal aspirate, VitekMS (bioMerieux) device, and MALDI-TOF MS method	77.80%
Clinical characteristics and risk factors for COVID-19-associated Candidemia	Observational	March 2020 and April 2021	Qatar	80		blood culture	72.60%
*Candida auris* Invasive Infections during a COVID-19 Case Surge	Case Report		USA	12		matrix-assisted laser desorption/ionization-time of flight (MALDI-ToF)	
Candidemia in COVID-19 treated with corticosteroids and tocilizumab	Case Report				Obesity, fever (38.6 °C), a saturation of percutaneous oxygen of 92%, respiratory rate of 24/min	blood culture	
Candidemia among Iranian Patients with Severe COVID-19 Admitted to ICUs	Original Article	November 2020 to January 2021	Iran	7		PCR, blood cultures, DNA Extraction, and Species Identification	100%
The challenge of preventing and containing outbreaks of multidrug-resistant organisms and *Candida auris* during the coronavirus disease 2019 pandemic: report of a carbapenem-resistant Acinetobacter baumannii outbreak and a systematic review of the literature	Systematic Review	September to early December					
COVID-19 and invasive fungal coinfections: A case series at a Brazilian referral hospital	Observational	February to December 2020	Brazil	4		clinical cultures	100%
Impact of the SARS-CoV-2 Pandemic in Candidaemia, Invasive Aspergillosis, and Antifungal Consumption in a Tertiary Hospital	Retrospective	April 2019 to March 2021	Spain	152		clinical cultures	27.9% and 57.7% in 2020 and 2021
COVID-19 and Candida duobushaemulonii superinfection: A case report	Case Report		Lebanon		acute respiratory distress syndrome (ARDS)	clinical cultures	
First Case of COVID-19 Positive *Candida auris* Fungemia in Turkey	Case Report	October 20	Turkey	1	acute respiratory distress syndrome (ARDS) and viral pneumonia	clinical cultures, VITEK MALDI TOF MS	100%
						confirmed by sequencing.	
Ominous combination: COVID-19 disease and *Candida auris* fungemia-Case report and review of the literature.	Case Report	-	-	1	fever, cough, and progressive shortness of breath, cytokine storm, liver, and acute kidney injury, new radiological infiltrates and worse condition	Blood culture obtained from central and peripheral lines	100%
Fungemia in COVID-19 ICU patients, a single medical center experience	Retrospective	March 2020–June 2020	USA	24	Prolonged mechanical ventilation support	Blood culture system	38%
						matrix-assisted laser desorption ionization time-of-flight mass spectrometry.	
Laboratory-based surveillance of *Candida auris* in Colombia, 2016–2020.	Original Article	March 2016 to December 2020.	Columbia	122	bloodstream infections, sepsis	Culture from skin, genito-urinary site. Biotyper MALDI-TOF	-
The Influence of Infection and Colonization on Outcomes in Inpatients With COVID-19: Are We Forgetting Something?	Observational	27 February 2020–26 February 2021	Spain	14	-	MALDI-TOF mass spectrometry, antimicrobial susceptibility tests	30.80%
Molecular Epidemiological Investigation of a Nosocomial Cluster of *C. auris*: Evidence of Recent Emergence in Italy and Ease of Transmission during the COVID-19 Pandemic	Original Article	July 2019 to May 2020	Italy	10	bloodstream infections and colonization	matrix-assisted laser desorption ionization-time of flight mass spectrometry (MALDI-TOF MS) and PCR amplification of the species-specific GPI protein-encoding genes	-
Axillary Digital Thermometers uplifted a multidrug-susceptible *Candida auris* outbreak among COVID-19 patients in Brazil	Case Report	1 December 20	Brazil	8	acute respiratory distress syndrome related to SARS-CoV-2 infection at admission.	Vitek 2 system, Culture, MALDI-TOF mass spectrometry	33.30%
Three *Candida auris* Case Reports from Istanbul, Turkey	Case Report	November 2020–January 2021	Turkey	1	Shortness of breathpneumoniaacute renal failure.	Catheter tip cultures MALDI-TOF Microflex LT/SH Smart MS confirmed by conventional and DNA methods.	33.30%
A High Frequency of *Candida auris* Blood Stream Infections in Coronavirus Disease 2019 Patients Admitted to Intensive Care Units, Northwestern India: A Case Control Study	Case Control Study	August 2020–January 2021	India	33		MALDI-TOF Bruker Biotyper. confirmed by amplification and sequencing of DNA.	
Candidemia in Coronavirus Disease 2019 (COVID-19) Patients: Incidence and Characteristics in a Prospective Cohort Compared With Historical Non–COVID-19 Controls	Observational	15 February 2020–30 June 2020	Italy	21	severe acute respiratory distress syndrome		57.10%
ICU-acquired Candidemia in COVID-19 Patients: An Experience from a Tertiary Care Hospital in Kerala, South India	Letter	July 5, 2020, to February 28, 2021	India	1			100%
Candida auris: A Latent Threat to Critically Ill Patients With Coronavirus Disease 2019	Letter	June to September 2020	Colombia	6	severe acute respiratory distress syndrome	matrix-assisted laser desorption/ionization time-of-flight mass spectrometry	100%

**Table 2:** Microbial co-infection in patients.

Microbial Species	Name of Organisms	Co-Infection
GRAM-POSITIVE BACTERIA	*Staphylococcus aureus* *Streptococcus pneumoniae* *Enterococcus faecium* *Enterococcus faecalis* *Coagulase negative-S.aureus*	Causes community/ventilator-associated pneumonia, necrotizing/cavitating pneumonia, sepsis shock, and Infective endocarditis [17,19,21,27]. Causes Hospital-acquired pneumonia [17]. Causes intra-abdominal infection, concurrent urinary tract infection, and bacteremia [17]. Causes urinary tract infection, intra-abdominal infection, and infective endocarditis in COVID 19 patients [17,21]. Causes bacteremia and sepsis [17,19].
GRAM-NEGATIVE BACTERIA	*Pseudomonas aeruginosa* *Klebsiella pneumoniae* *Escherichia coli* *Acinetobacter baumannii* *Stenotrophomonas maltophilia* *Haemophilus influenzae*	Causes hospital/ventilator-associated pneumonia, bacteremia, urinary tract infection, and lower respiratory tract infection with bronchiectasis in severely ill COVID patients [17]. Causes ventilator-associated pneumonia and concurrent urinary tract infection [17]. Causes bacteremia, concurrent urinary tract infection, and intra-abdominal infection with *E. faecalis* and *E. faecium* [17]. Causes ventilator-associated pneumonia in patients with comorbidities like diabetes, and increased stay in ICU [17,22]. Causes ventilator-associated pneumonia and nosocomial infection in immunosuppressed COVID patients [17,23]. Causes community-acquired pneumonia [17].
ATYPICAL BACTERIA	*Mycoplasma pneumoniae* *Moraxella catarrhalis* *Mycobacterium tuberculosis*	Causes interstitial pneumonia and exacerbation COVID symptoms [17]. Community-acquired interstitial pneumonia [17].Causes caseating granulomas and activation of latent T.B. due to immune dysfunction in COVID infection [35].
FUNGI	*Candida auris*	Persistent fungemia in immunocompromised individuals, urinary tract infection Eye, ear, and nose infections associated with COVID patients having co-morbidities [31,32].
	*Candida albicans* *Candida dubliniensis* *Candida glabrata* *Candida krusei* *Candida parapsilosis*	Causes oropharyngeal candidiasis, oropharyngeal thrush with erythematous inflammation, and infective endocarditis. Invasive in immunocompromised individuals causing infection in the blood; candidemia. Associated with ventilation, intubation and urine catheterization [21,31,32].
	*Aspergillus fumigatus* *Aspergillus flavus*	Causes tracheobronchitis, necrotizing pneumonia, and bronchial wall ulceration in COVID 19 patients on the ventilator [32].
VIRUSES	*Respiratory Syncytial virus* *Influenza A/B virus* *Human metapneumovirus* *rhino/enterovirus* *Parainfluenza* *Epstein-Barr virus*	Causes cold/ flu-like symptoms (cough, sore throat, fever, rhinorrhea, and dyspnea) but can exacerbate COVID symptoms causing acute respiratory distress syndrome [33,34]. Causes Infectious Mononucleosis (I.M.) and splenomegaly [27].
	*H.I.V.* *Hepatitis B/C virus*	Leads to a decrease in CD4+ T cells and immunosuppression in COVID patients [19]. Causes liver impairment and systemic inflammation [19].

**Table 3:** Opportunistic mycoses in COVID-19 infection.

Invasive Fungal Infection	Fungi Species	Name of Organism
Invasive candidiasis	*Candida* spp.	*Candida albicans* *Candida tropicalis* *Candida parapsilosis* [36,40,41,42,43,44].
COVID-associated pulmonary aspergillosis [CAPA]	*Aspergillus* spp.	*Aspergillus niger* *Aspergillus fumigatus* *Aspergillus lentulus* *Aspergillus terreus* *Aspergillus trinoterreus* [36,37,38,39,40,41,42,43,44]
Mucormycosis	*Rhizopus* spp.	*Rhizopus stolonifera* *Rhizopus microspores* [42]
Cryptococcosis	*Cryptococcus* spp.	*Cryptococcus neoformans* [44]
Histoplasmosis	*Histoplasmosis* spp.	*Histoplasmosis capsulatum* [44]

**Table 4:** Risk factors of *C. auris* in COVID-19 patients.

Clinical Risk Factors	Non-Clinical Risk Factors	Medication-Related Risk Factors
Diabetes Mellitus [4,9,71,81,82,83]	Prolonged ICU stay [51,71]	Corticosteroids [5,9,51,71,81,82,83,84]
Renal Failure [81]	Age > 60 [78]	Catheter venous, Foley catheter [5,9,81,84]
Chronic Kidney Disease [4,9,82]	Male sex [78]	Mechanical Ventilation and/or intubation [5,9,71,84,85]
Ventilator-associated pneumonia [71]	Obesity [83]	Incorrect use of PPE [82]
Hypertension [71]		Broad spectrum antibiotics [5,9,51,71,81,84)
Invasive hemodynamic monitoring		Interleukin-6 receptor inhibitors [71]
Lymphopenia [71]		Tocilizumab [85]
Cardiovascular diseases [71,83]		Colonized digital thermometers [5]
Urinary tract infections [71]		Dialysis [84]

## Data Availability

Data sharing does not apply to this article as no datasets were generated or analyzed during the current study.
